# Global trends in cervical spondylosis research: a bibliometric analysis based on the Web of Science

**DOI:** 10.3389/fneur.2025.1541459

**Published:** 2025-04-30

**Authors:** Lin Jiang, Yu Xu, Zhijun Yang, Penghui Li, Youkang Dong, Guangzhi Yang

**Affiliations:** ^1^Second Clinical Medical College, Yunnan University of Chinese Medicine, Kunming, China; ^2^Department of Tuina, The First Affiliated Hospital of Yunnan University of Chinese Medicine, Kunming, China; ^3^Department of Rehabilitation, Lincang Municipal Hospital of Chinese Medicine, Lincang, China

**Keywords:** cervical spondylosis, bibliometrix, network analysis, research frontiers, Web of Science

## Abstract

**Objective:**

We aim to analyze the development trends in cervical spondylosis research and guide future studies. Cervical spondylosis, a standard neck disorder characterized by pain, nerve compression, and spondylosis, is highly prevalent, particularly among older individuals, due to the extensive use of electronic devices. Since treatment options are limited and surgery is considered a last resort, it is crucial to explore the current research status and identify areas for further investigation.

**Method:**

We conducted a bibliometric analysis of academic articles on cervical spondylosis published between 1980 and 2022. The analysis involved utilizing the Web of Science database and employing R software and a VOS viewer.

**Results:**

Our analysis revealed that neurosciences and neurology were the primary research focus, with participation from 62 countries. China had the highest number of publications, while the USA received the most citations. The Rothman Institute emerged as the most cited institution in neck pain research. The journal “Spine” had the highest publication count. Among authors, Mummaneni P. V. was the most cited, and Liu H. had the highest number of publications. The keyword “Spine” was the most frequently used.

**Conclusion:**

Our bibliometric study summarized the current research status of cervical spondylosis. Further investigations are warranted in diagnosis, treatment, prevention, non-surgical interventions, and rehabilitation. Promising areas of interest include artificial cervical discs, gene therapy, and stem cell therapy. Our study provides a framework for enhancing cervical spondylosis’s diagnosis and treatment by addressing existing literature gaps.

## 1 Introduction

Cervical spondylosis (CS) is a progressive degenerative disorder of the cervical spine, presenting with a wide range of symptoms, including neck pain, shoulder and back pain, upper limb numbness and discomfort, headache, dizziness, nausea, vomiting, gastrointestinal disturbances, blurred vision, and tinnitus (1). The etiology of CS is multifactorial and closely linked to prolonged neck strain due to excessive electronic device use, forward head posture, increased mechanical stress on the cervical spine, and degenerative changes caused by the straightening of physiological cervical curvature (2, 3). Treatment options for CS remain limited and typically involve a combination of physical therapy, pharmacological interventions, and surgical procedures. Surgery is generally recommended for severe cases or when conservative treatments fail; however, surgical interventions carry inherent risks and potential complications (4). CS has become a prevalent global health concern, with a particularly high incidence among the elderly. Studies indicate that approximately 85% of individuals over the age of 60 suffer from CS, and its prevalence is increasing among younger populations (5).

Despite the extensive research on CS, a comprehensive analysis summarizing research trends and advancements in this field remains lacking. A bibliometric analysis is a valuable tool for systematically assessing the existing literature, identifying key research hotspots, and highlighting knowledge gaps. Such an approach enables researchers and clinicians to gain deeper insights into the evolution of CS studies, highlight potential directions for future research, and refine clinical strategies for diagnosis and treatment.

This study examines CS-related research published between 1980 and 2022—a period marked by significant advancements from fundamental research to clinical applications. This timeframe was chosen to capture the evolution of CS research, particularly in light of the increasing use of computers and mobile devices, which has profoundly influenced the epidemiology of the condition. Furthermore, this period encompasses critical technological and medical developments that have shaped contemporary approaches to CS management.

Bibliometric analysis provides a robust framework for quantifying and visualizing academic contributions, identifying influential authors, institutions, and journals, and recognizing emerging trends. By leveraging bibliometric tools, this study aims to present a comprehensive overview of CS research, assisting scholars and clinicians in navigating the vast body of literature and guiding future studies toward critical yet underexplored areas.

## 2 Methods

### 2.1 Data source and search strategy

We conducted a literature search in the Web of Science (WOS) Core Collection Complete database on April 7, 2023. The WOS database was selected for its extensive coverage of high-impact journals and its widespread application in bibliometric research. The search was performed using the following query: TS = (“cervical spondylosis” OR “cervical osteoarthritis” OR “cervical degenerative disc disease” OR “cervical disc degeneration” OR “cervical spinal stenosis” OR “neck arthritis” OR “neck osteoarthritis” OR “neck degenerative disc disease” OR “neck disc degeneration” OR “neck spinal stenosis”), resulting in the retrieval of 2,217 relevant documents.

**Figure 1:**
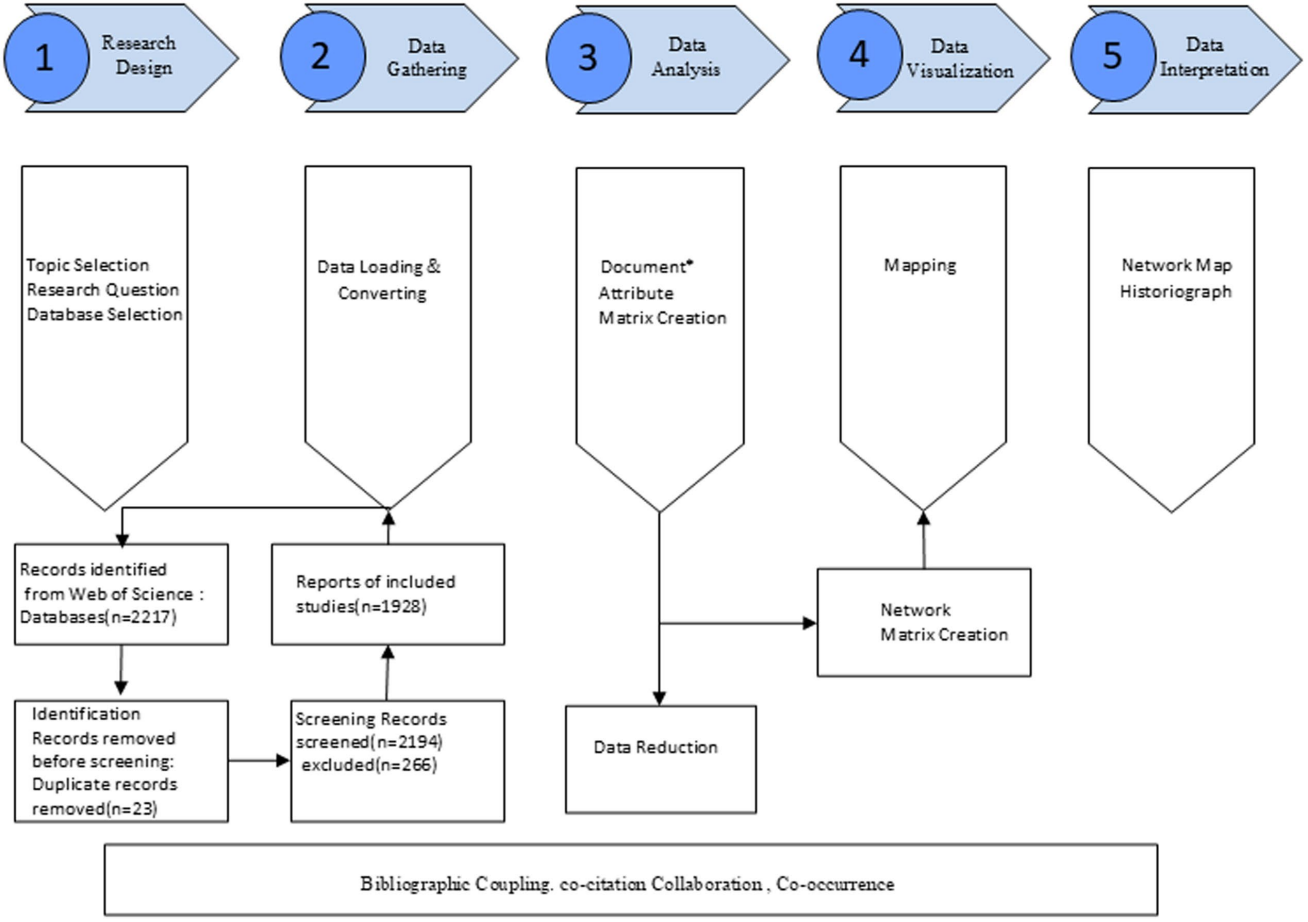
Workflow of bibliometric analysis on cervical spondylosis research.

All records were saved as “Plain Text Files,” including “Full Records and Cited References.” Following the bibliometric analysis framework proposed by Aria and Cuccurullo (6), we employed a structured five-step approach: research design, data collection, analysis, visualization, and interpretation (7–9). A summary of this methodological workflow is presented in Figure 1.

### 2.2 Data screening and inclusion criteria

To enhance data quality and ensure the reliability of our analysis (9), we applied a document-type filter in WOS, including only research articles and review papers while excluding conference proceedings, book chapters, and editorials. Additionally, we restricted the analysis to English-language publications, as English is the predominant language in high-impact scientific literature. However, we acknowledge that this decision may introduce a language bias, which is discussed as a limitation in our study.

1. Automatic deduplication: We utilized WOS’s built-in tools to remove duplicate records.
2. Title and abstract screening: Two independent reviewers manually screened article titles and abstracts to exclude irrelevant studies not focused on CS.
3. Final inclusion based on predefined criteria: We applied predefined inclusion and exclusion criteria, selecting studies that directly investigated CS-related topics, such as epidemiology, pathogenesis, diagnosis, treatment, and prognosis. Studies unrelated to CS, or those with insufficient data, were excluded. This screening process refined our dataset to 1,928 final articles for analysis.

### 2.3 Justification of the time range (1980–2022)

The study period of 1980–2022 was chosen to capture the long-term evolution of CS research. The 1980s marked a pivotal era in spinal disease research, driven by the advent of advanced imaging techniques such as MRI and CT scans, as well as the development of evolving treatment strategies. Furthermore, a significant increase in CS-related publications during this period makes it an appropriate timeframe for analyzing research trends.

### 2.4 Data processing and analysis

All retrieved records were imported into Biblioshiny Web and converted into Bibliometrix RData and Excel formats for further analysis. Key bibliometric indicators, including publication trends, author collaborations, keyword collaboration, and citation patterns, were examined. To identify major research areas and emerging trends in CS studies, visualization techniques such as network mapping and thematic clustering were applied.

Bradford’s Law, a fundamental bibliometric principle, describes the distribution of articles across journals within a given research field (10). This principle observes that the number of articles published in a field is inversely proportional to their rank when journals are sorted by productivity. Widely employed in bibliometric research, Bradford’s Law helps identify influential sources and assess the distribution of publications. In the following sections, we will present the results of data analysis and visualization, providing insights into publication distribution within the field of CS research. These findings serve as a valuable reference for future studies and inform decision-making in both academic and clinical contexts.

## 3 Results

The bibliometric analysis provides a comprehensive overview of CS research development. This study examined 1,928 documents published between 1980 and 2022. The analysis covered multiple dimensions, including authorship, journals, research topics, keywords, countries, and institutions, offering insights into the field’s evolution.

### 3.1 Publication trends

**Figure 2:**
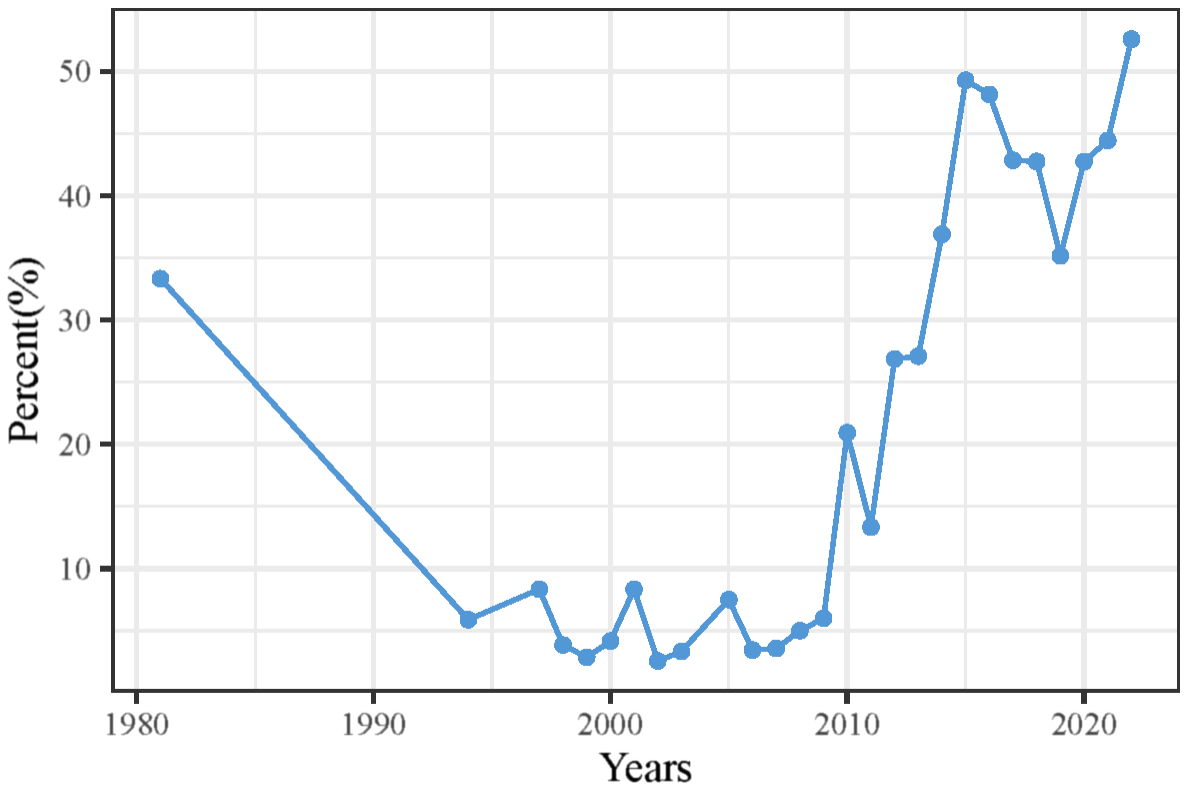
Publication trends in cervical spondylosis research from 1980 to 2022.

Figure 2 illustrates the annual publication trend, starting with five papers in 1980 and demonstrating steady growth. After 2015, the number of CS-related publications increased significantly, reaching 173 in 2022, with an average annual growth rate of 14.76%. Primary statistics of cervical spondylosis-related literature derived from bibliometric analysis.

Supplementary Table 1 presents key bibliometric indicators for the 1,928 papers retrieved from the WOS SCI ALL database between 1980 and 2022. On average, 46 CS-related papers were published annually over the 42-year period, with each paper receiving an average of 21.38 citations. Additionally, the dataset includes a total of 3,492 unique author keywords, reflecting the diversity of research topics in the field.

### 3.2 Research fields

CS research in the Web of Science (WOS) database spans 24 research areas, underscoring the interdisciplinary nature of this field. Since 1982, CS-related studies have expanded across diverse disciplines.

The top 10 most productive research areas, accounting for 88.20% of total publications, include:

1. Neurosciences & Neurology.
2. Orthopedics.
3. Surgery.
4. General & Internal Medicine.
5. Research & Experimental Medicine.
6. Radiology, Nuclear Medicine & Medical Imaging.
7. Rheumatology.
8. Rehabilitation.
9. Engineering.
10. Integrative & Complementary Medicine.

**Figure 3:**
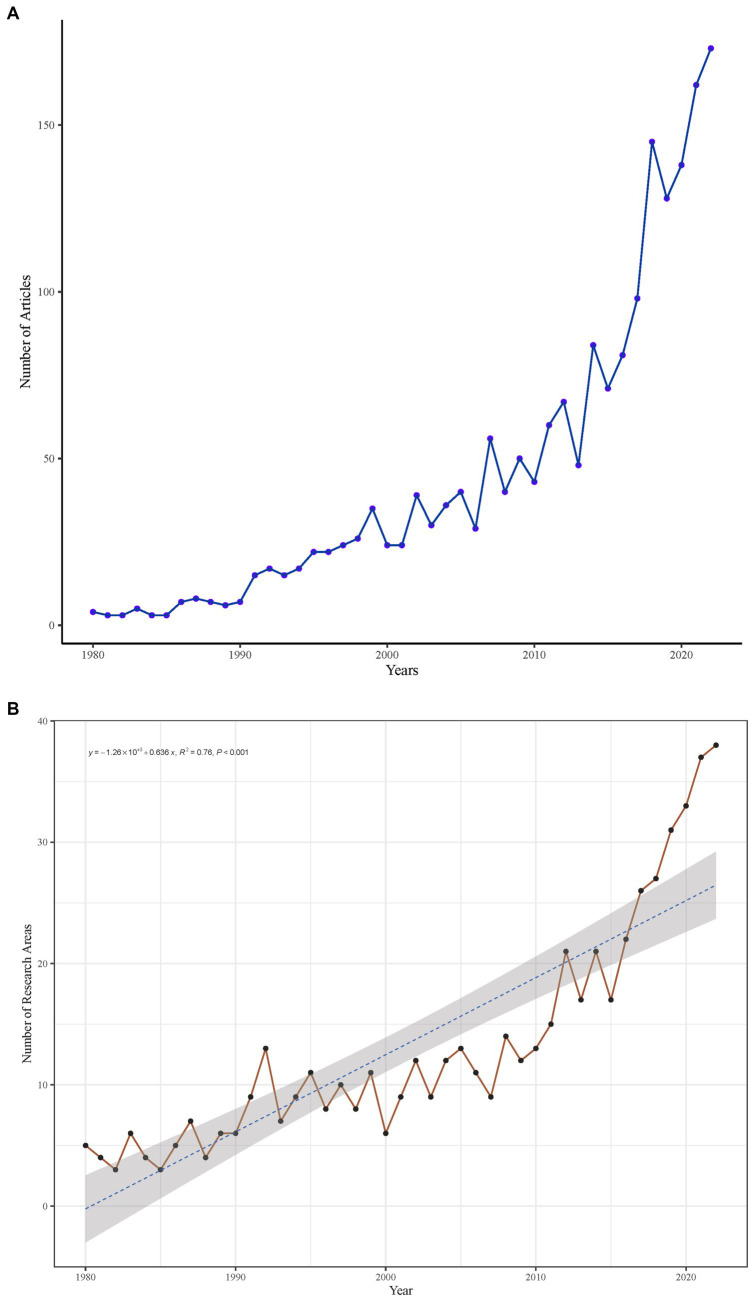
**(A)** Distribution of research areas in cervical spondylosis-related literature. **(B)** Temporal evolution of the top 10 most productive research areas from 1980 to 2022.

Figure 3A presents the distribution of research areas, while Figure 3B illustrates the evolution of CS research focus over time. Neurosciences and neurology remain the dominant discipline (1980–2022), producing the highest number of publications in 2022.

### 3.3 Country and institutional contributions

CS research has been conducted in 62 countries. The top five contributing countries, in terms of publication volume, are:

1. China (552 papers).
2. United States (510 papers).
3. Japan (198 papers).
4. India (70 papers).
5. South Korea (69 papers).

**Figure 4:**
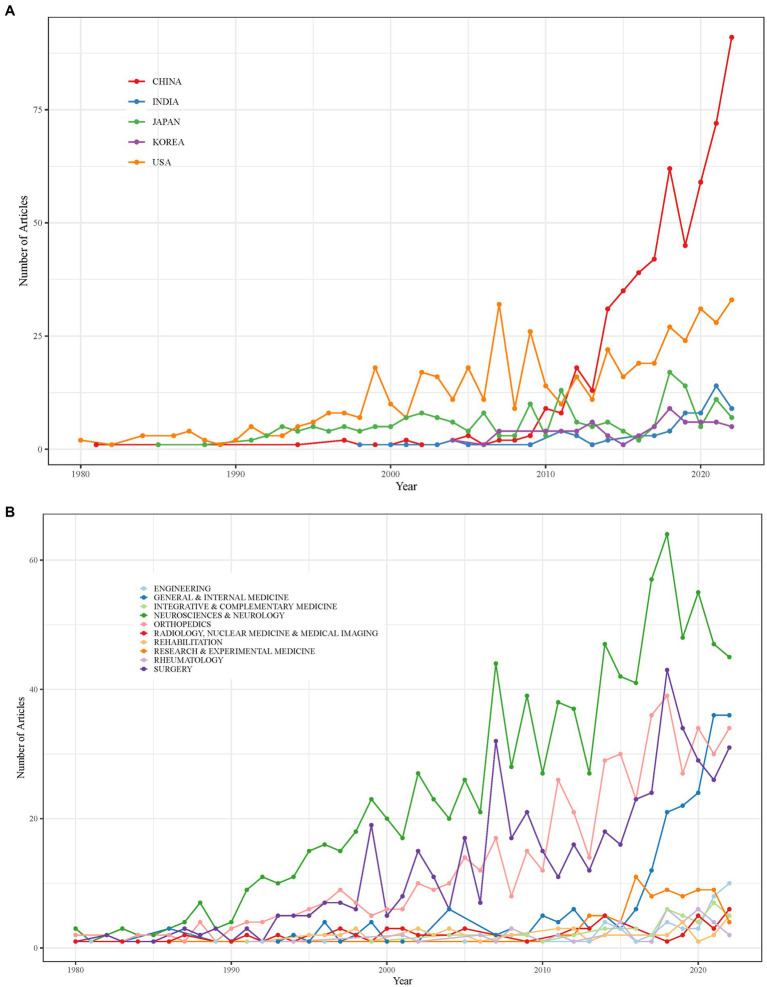
**(A)** Annual scientific production in the top 5 countries contributing to cervical spondylosis research. **(B)** Annual proportion of publications from China in global cervical spondylosis literature.

Since 2014, China’s scientific output in CS research has increased substantially, surpassing that of the United States (Figure 4A). No CS-related publications from China were recorded between 1981 and 1994; however, its research contributions grew sharply after 2012, accounting for 52.60% of global CS research output in 2022 (Figure 4B).

**Figure 5:**
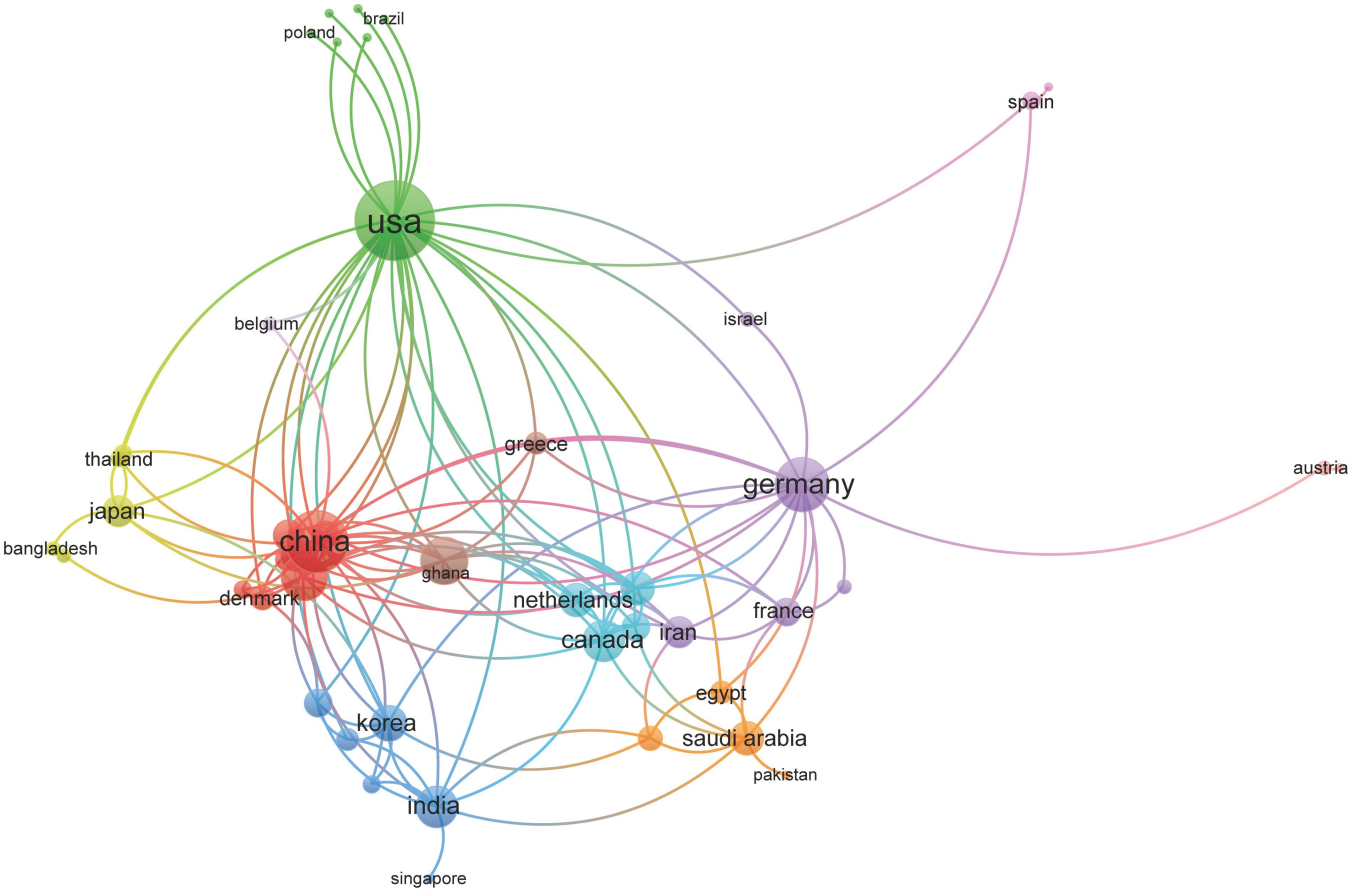
Map of inter-nation research collaboration.

International collaboration networks further highlight the global impact of CS research. Figure 5 illustrates global research collaboration patterns, with the United States leading in international partnerships (31 collaborations), followed by China (21 collaborations), Germany (17 collaborations), the UK (14 collaborations), and Australia (13 collaborations).

## 4 Discussion

### 4.1 Research growth and emerging trends

The sharp rise in CS-related publications, particularly after 2015, highlights the increasing recognition of CS as a critical public health concern. This surge reflects accelerated research activity, with an average annual growth rate of 14.76%. Advances in diagnostic imaging techniques (e.g., MRI, CT scans), biomechanics, and minimally invasive surgical procedures have likely contributed to this expansion in research output.

### 4.2 Country-specific contributions and research leadership

China and the United States have emerged as the leading contributors to CS research. Notably, China had no recorded research activity in CS before 1994, but its output has grown substantially since then. This increase aligns with government-funded initiatives focusing on spinal disorders and orthopedic research. Furthermore, the extensive international collaboration network involving the United States, Germany, and the United Kingdom underscores the global significance of CS research.

### 4.3 Comparison with previous bibliometric studies

When compared with previous bibliometric analyses on spinal disorders (e.g., lumbar disc herniation and scoliosis), the publication trends in CS exhibit similar patterns, with neuroscience and neurology being the predominant disciplines. However, unlike research on lumbar disorders, which places a strong emphasis on biomechanics, CS research more prominently integrates elements of neurology, rheumatology, and rehabilitation.

### 4.4 Most influential papers

We categorized the essential papers using their local citation score (LCS) and global citation score (GCS), which measure citation impact within and across fields, respectively (11). We assessed each paper’s significance in the CS field using LCS and GCS. Papers with high LCS and low GCS were deemed highly significant within CS, while those with high GCS and low LCS were considered universally significant (12, 13) (Supplementary Tables 2, 3). Alan S. Hilibrand authored the top paper in LCS (and ranked first in GCS) in 1999. It investigated the occurrence, frequency, and X-ray progression of adjacent segment degeneration following anterior cervical fusion, specifically focusing on new radiculopathy or myelopathy near previously fused motion segments (14). Alan S. Hilibrand’s paper significantly impacted the CS field and remains highly cited and influential. The second most impactful LCS paper in CS (ranked third in GCS) compared the effectiveness of anterior cervical fusion and posterior cervical foraminotomy for treating single-level cervical radiculopathy, evaluating the PRESTIGE ST cervical disc system prosthesis.

Furthermore, a large, multicenter, randomized controlled study design was employed to bolster the reliability of the research findings (15). The paper ranked third in LCS (second in GCS) proposed strategies to prevent and manage adverse effects and complications of anterior cervical discectomy and fusion (ACDF), emphasizing the importance of prompt identification and management of ACDF-related complications (16). The findings are significant for future research in this area. The fourth-ranked LCS paper (also fourth in GCS) systematically evaluated and compared the effectiveness of various studies on using anterior cervical plates in ACDF surgeries. The results indicated that anterior cervical plates could significantly improve bone graft fusion rates and reduce complication rates (17), providing doctors with more effective and safer treatment options and ultimately enhancing patient outcomes and quality of life.

The fifth-ranked LCS article (eighth in GCS) introduces the vertebral body ratio (VBR) method for determining cervical spinal canal stenosis. This reliable method is independent of technical factors and provides ratios for male and female control groups, explaining its radiological principles. Results show that the VBR method accurately detects cervical spinal canal stenosis. Compared to traditional methods, it is more precise and reliable (18). The sixth-ranked LCS research focuses on diagnosing and treating CS and neck pain, identifying knowledge gaps and areas needing further research, and guiding future clinical practice and scientific studies (19).

The seventh-ranked LCS research offers a long-term follow-up study assessing the long-term impacts of surgical treatment on cervical spondylotic myelopathy patients, identifying factors influencing surgical outcomes. It also provides insights into individualized treatment, aiding doctors in predicting surgical treatment efficacy (20). The latter paper, a prospective, non-blinded study involving 541 patients across 32 centers, compared the effectiveness of Prestige disc replacement and ACDF in treating cervical degenerative disease and assessed their long-term effects. At 36 and 60 months post-surgery, no statistically significant differences were observed in cervical range of motion, cervical height, adjacent segment degeneration, work status, income, or education between patients who underwent Prestige disc replacement and those who underwent ACDF (21). These findings offer clinicians more insights into treating cervical degenerative disease, aiding doctors in selecting suitable treatment options. The ninth-ranked LCS paper, fifth in GCS, examined empirical data on the therapeutic effects and potential long-term impacts of front neck discectomy and cervical spine fusion surgery on myelopathy and radiculopathy caused by cervical degenerative disease. The article also explored whether postoperative cervical deformity promotes adjacent disc-level degeneration, providing clinicians with additional information for post-surgery follow-up and treatment planning (22). LCS ranks the 10th study, introducing a novel artificial cervical joint, the NFCJ, designed to mimic human cervical motion and preserve a spinal range of motion. This study also investigated the effects of disc replacement surgery on adjacent segments, finding that it preserves motion rather than fusing the degenerative spine. By monitoring postoperative patients and assessing disability and reoperation rates for adjacent segment disease, the study offers a crucial reference for clinical practice (23). The sixth most influential GCS article evaluates the long-term effectiveness of the original open-door laminoplasty for cervical myelopathy, presenting valuable clinical data. Based on clinical assessments and imaging results, the study concludes that this surgical method yields lasting positive outcomes. It also identifies associated problems and risks, offering insights for future surgical technique improvements and refined treatment plans (24). Although not among the LCS top 10, this document holds significant importance. Despite ranking 7th in GCS, it missed the LCS top 10. The paper explores the long-term effects of double-door laminoplasty on cervical spondylotic myelopathy, showing that it enhances neurological function and prevents late deterioration due to cervical degeneration and spinal cord injury (25). The 9th and 10th GCS-ranked papers did not make the LCS top 10. The 9th reviews multilevel cervical spondylotic myelopathy treatments, surgical techniques, and related studies. The authors compare surgical methods, highlight the pros and cons, and provide valuable recommendations for clinicians (26). The latter paper conducted a retrospective cohort study using population-based data to assess cervical spine surgery’s complications and mortality. It explored the correlations between age, primary diagnosis, surgical approach, and various outcomes. Additionally, the authors crafted an algorithm utilizing International Classification of Diseases-Ninth Revision Clinical Modification codes to define degenerative cervical spine disease and its complications, serving as a framework for future research (27). Besides the 5th most influential LCS paper on the vertebral body ratio method for cervical spinal stenosis, the 6th paper explored cervical spine disease, neck pain diagnoses, and treatment evidence. Most other literature focused on surgical treatments for various CS types. LCS identified six of the top 10 influential papers overlapping with GCS papers, while the rest emphasized surgical treatment effectiveness. Age, gender, primary diagnosis, and surgery type significantly impact surgical outcomes.

Carrier et al. (28) stated that degenerative cervical disease is the primary reason for cervical spine surgery. Wada et al. (29) found that spinal cord function loss in cervical spine disease is mainly due to irreversible white matter damage, often caused by long-term spinal cord compression. This damage often remains after surgery, leading to poor neurological recovery. Furthermore, nonunion and perioperative complications frequently contribute to unsatisfactory surgical outcomes.

Moreover, Exelby (30) explored the potential of the Mulligan concept in evidence-based practice and suggested future paths for joint mobilization therapy in treating CS. Other critical research areas, including prevention, non-surgical treatment, and rehabilitation of cervical spine disease, have garnered significant attention. As medical technology advances, research on new treatment methods and technologies, such as implantable cervical artificial intervertebral discs (31), gene therapy (32), and stem cell therapy (33), has become crucial and deserves further investigation.

In summary, future research on CS should focus on practical, safe, and individualized treatment plans based on comprehensive patient evaluations to enhance disease management. By incorporating these factors into clinical practice, health.

### 4.5 Study limitations

This study exclusively analyzed English-language publications indexed in the Web of Science (WOS) database, which may introduce language and database bias. This limitation should be considered when interpreting the results, as it may exclude relevant research published in other languages or indexed in different databases. Future bibliometric analyses could enhance comprehensiveness by incorporating additional databases (e.g., PubMed, Scopus) and including non-English literature to provide a more holistic perspective on CS research.

## 5 Conclusion

This bibliometric analysis examined the development trends of CS research from 1980 to 2022, revealing a substantial increase in research output in recent years. The findings indicate that the United States, China, the United Kingdom, and South Korea have made significant contributions to CS research. Among the top research institutions identified were the Rothman Institute and the University of California, San Francisco. Prominent researchers in the field include Mummaneni P. V., Holly L. T., and Kaiser M. G. The scope of CS research continues to expand, with evolving methodologies shaping the field. Based on emerging research themes, we anticipate that personalized assessment and treatment management approaches will become key focal points in future CS studies. Additionally, advancements in data analysis techniques and patient-centered treatment strategies hold significant potential to enhance diagnostic accuracy and improve treatment outcomes for cervical spondylosis.

## Data Availability

The raw data supporting the conclusions of this article will be made available by the authors, without undue reservation.

## Supplementary material

SUPPLEMENTARY TABLE 1 Primary statistics of cervical spondylosis-related literature derived from bibliometric analysis.

SUPPLEMENTARY TABLE 2 Top 10 papers with the highest local citation score.

SUPPLEMENTARY TABLE 3 Top 10 papers with the highest global citation score.

## References

[1] Leung KKY Chu EC-P Chin WL Mok STK Chin EWS. Cervicogenic visual dysfunction: an understanding of its pathomechanism. Med Pharm Rep. (2023) 96:16–9. doi: 10.15386/mpr-2507, PMID: 36818319 PMC9924804

[2] Chu ECP Lo FS Bhaumik A. Plausible impact of forward head posture on upper cervical spine stability. J Fam Med Prim Care. (2020) 9:2517–20. doi: 10.4103/jfmpc.jfmpc_95_20, PMID: 32754534 PMC7380784

[3] Koh MJ Park SY Woo YS Kang SH Park SH Chun HJ . Assessing the prevalence of recurrent neck and shoulder pain in Korean high school male students: a cross-sectional observational study. Korean J Pain. (2012) 25:161–7. doi: 10.3344/kjp.2012.25.3.161, PMID: 22787546 PMC3389320

[4] Theodore N. Degenerative cervical spondylosis. N Engl J Med. (2020) 383:159–68. doi: 10.1056/NEJMra2003558 32640134

[5] Gao Q-Y Wei F-L Zhu K-L Zhou C-P Zhang H Cui W-X . Clinical efficacy and safety of surgical treatments in patients with pure cervical radiculopathy. Front Public Health. (2022) 10:892042. doi: 10.3389/fpubh.2022.892042, PMID: 35910906 PMC9330161

[6] Aria M Cuccurullo C. Bibliometrix: an R-tool for comprehensive science mapping analysis. J Informet. (2017) 11:959–75. doi: 10.1016/j.joi.2017.08.007

[7] Zupic I Čater T. Bibliometric methods in management and organization. Organ Res Methods. (2015) 18:429–72. doi: 10.1177/1094428114562629

[8] Xu Y Yang Y Chen X Liu Y. Bibliometric analysis of global NDVI research trends from 1985 to 2021. Remote Sens. (2022) 14:3967. doi: 10.3390/rs14163967, PMID: 40053772

[9] Secinaro S Brescia V Calandra D Biancone P. Employing bibliometric analysis to identify suitable business models for electric cars. J Clean Prod. (2020) 264:121503. doi: 10.1016/j.jclepro.2020.121503

[10] Brookes BC. Sources of information on specific subjects by S.C. Bradford. J Inf Sci. (1985) 10:173–5. doi: 10.1177/016555158501000406, PMID: 40182056

[11] Garfield E. Citation indexes for science: a new dimension in documentation through association of ideas. Science. (1955) 122:108–11. doi: 10.1126/science.122.3159.108, PMID: 14385826

[12] Bornmann L Daniel H. What do citation counts measure? A review of studies on citing behavior. J Doc. (2008) 64:45–80. doi: 10.1108/00220410810844150

[13] Waltman L. A review of the literature on citation impact indicators. J Informet. (2016) 10:365–91. doi: 10.1016/j.joi.2016.02.007

[14] Hilibrand AS Carlson GD Palumbo MA Jones PK Bohlman HH. Radiculopathy and myelopathy at segments adjacent to the site of a previous anterior cervical arthrodesis. J Bone Joint Surg. (1999) 81:519–28. doi: 10.2106/00004623-199904000-00009, PMID: 10225797

[15] Huddleston PM. Clinical and radiographic analysis of cervical disc arthroplasty compared with allograft fusion: a randomized controlled clinical trial. Yearb Orthop. (2008) 2008:219–20. doi: 10.1016/S0276-1092(08)79337-4 17355018

[16] Fountas KN Kapsalaki EZ Nikolakakos LG Smisson HF Johnston KW Grigorian AA . Anterior cervical discectomy and fusion associated complications. Spine. (2007) 32:2310–7. doi: 10.1097/BRS.0b013e318154c57e, PMID: 17906571

[17] Kaiser MG Haid RW Subach BR Barnes B Rodts GE. Anterior cervical plating enhances arthrodesis after discectomy and fusion with cortical allograft. Neurosurgery. (2002) 50:229–38. doi: 10.1227/00006123-200202000-00001, PMID: 11844257

[18] Pavlov H Torg JS Robie BJ Jahre C. Cervical spinal stenosis: determination with vertebral body ratio method. Radiology. (1987) 164:771–5. doi: 10.1148/radiology.164.3.3615879, PMID: 3615879

[19] Binder AI. Cervical spondylosis and neck pain. BMJ. (2007) 334:527–31. doi: 10.1136/bmj.39127.608299.80, PMID: 17347239 PMC1819511

[20] Ebersold MJ Pare MC Quast LM. Surgical treatment for cervical spondylitic myelopathy. J Neurosurg. (1995) 82:745–51. doi: 10.3171/jns.1995.82.5.0745, PMID: 7714597

[21] Burkus JK Haid RW Traynelis VC Mummaneni PV. Long-term clinical and radiographic outcomes of cervical disc replacement with the prestige disc: results from a prospective randomized controlled clinical trial. J Neurosurg Spine. (2010) 13:308–18. doi: 10.3171/2010.3.SPINE09513, PMID: 20809722

[22] Katsuura A Hukuda S Saruhashi Y Mori K. Kyphotic malalignment after anterior cervical fusion is one of the factors promoting the degenerative process in adjacent intervertebral levels. Eur Spine J. (2001) 10:320–4. doi: 10.1007/s005860000243, PMID: 11563618 PMC3611517

[23] Wigfield C Gill S Nelson R Langdon I Metcalf N Robertson J. Influence of an artificial cervical joint compared with fusion on adjacent-level motion in the treatment of degenerative cervical disc disease. J Neurosurg Spine. (2002) 96:17–21. doi: 10.3171/spi.2002.96.1.0017, PMID: 11795709

[24] Chiba K Ogawa Y Ishii K Takaishi H Nakamura M Maruiwa H . Long-term results of expansive open-door laminoplasty for cervical myelopathy-average 14-year follow-up study. Spine. (2006) 31:2998–3005. doi: 10.1097/01.brs.0000250307.78987.6b, PMID: 17172996

[25] Seichi A Takeshita K Ohishi I Kawaguchi H Akune T Anamizu Y . Long-term results of double-door laminoplasty for cervical stenotic myelopathy. Spine. (2001) 26:479–87. doi: 10.1097/00007632-200103010-00010, PMID: 11242374

[26] Ratliff JK Cooper PR. Cervical laminoplasty: a critical review. J Neurosurg Spine. (2003) 98:230–8. doi: 10.3171/spi.2003.98.3.0230, PMID: 12691377

[27] Wang MC Chan L Maiman DJ Kreuter W Deyo RA. Complications and mortality associated with cervical spine surgery for degenerative disease in the United States. Spine. (2007) 32:342–7. doi: 10.1097/01.brs.0000254120.25411.ae, PMID: 17268266

[28] Carrier CS Bono CM Lebl DR. Evidence-based analysis of adjacent segment degeneration and disease after ACDF: a systematic review. Spine J. (2013) 13:1370–8. doi: 10.1016/j.spinee.2013.05.050, PMID: 23891293

[29] Hurtado I García-Sempere A Peiró S Rodríguez-Bernal C Sanfélix-Genovés J Sanfélix-Gimeno G. Trends and geographical variability in osteoporosis treatment after hip fracture: a multilevel analysis of 30,965 patients in the region of Valencia, Spain. J Bone Miner Res. (2020) 35:1660–7. doi: 10.1002/jbmr.4028, PMID: 32297654 PMC9328445

[30] Exelby L. The Mulligan concept: its application in the management of spinal conditions. Man Ther. (2002) 7:64–70. doi: 10.1054/math.2001.0435, PMID: 12374089

[31] Peng Z Hong Y Meng Y Liu H. A meta-analysis comparing the short- and mid- to long-term outcomes of artificial cervical disc replacement (ACDR) with anterior cervical discectomy and fusion (ACDF) for the treatment of cervical degenerative disc disease. Int Orthop. (2022) 46:1609–25. doi: 10.1007/s00264-022-05318-z, PMID: 35113188

[32] Richens J McGill PE. The spondyloarthropathies. Baillière’s Clin Rheumatol. (1995) 9:95–109. doi: 10.1016/S0950-3579(05)80147-5, PMID: 7728892

[33] Shnayder NA Ashhotov AV Trefilova VV Nurgaliev ZA Novitsky MA Vaiman EE . Cytokine imbalance as a biomarker of intervertebral disk degeneration. Int J Mol Sci. (2023) 24:2360. doi: 10.3390/ijms24032360, PMID: 36768679 PMC9917299

