# Supplementary material for: Global trends in cervical spondylosis research: a bibliometric analysis based on the Web of Science
Source: Front Neurol. 2025 Apr 30;16:1541459. doi: 10.3389/fneur.2025.1541459 (PMC12075215; doi:10.3389/fneur.2025.1541459)
Supplement: SUPPLEMENTARY TABLE 1 — Primary statistics of cervical spondylosis-related literature derived from bibliometric analysis. [file Data_Sheet_1.pdf]

**Mian information**	**Description**	**Value**
Documents	Total number of documents	1,928
Sources	The frequency distribution of sources as journals, books, etc.	543
Timespan	Years of publication	1980 :2022
References	Total number of references	31,136
Author's Keywords (DE)	Total number of author's keywords	3,492
Keywords Plus (ID)	Total number of phrases that frequently appear in the title of an article's references	2,762
Authors	Total number of authors	7,282
Author Appearances	The authors' frequency distribution	10,300
Authors of single-authored docs	The number of single authors per articles	87
Documents per Author	Average number of authors in each document	0.265
Co-Authors per Doc	Average number of co-authors in each document	5.34
Average citations per doc	Average number of citations in each document	21.38
