# Supplementary material for: Global trends in cervical spondylosis research: a bibliometric analysis based on the Web of Science
Source: Front Neurol. 2025 Apr 30;16:1541459. doi: 10.3389/fneur.2025.1541459 (PMC12075215; doi:10.3389/fneur.2025.1541459)
Supplement: SUPPLEMENTARY TABLE 3 — Top 10 papers with the highest global citation score. [file Data_Sheet_3.pdf]

**Document**	**DOI**	**Year**	**Local Citations**	**Global Citations**
Hilibrand As, 1999, J Bone Joint Surg Am	10.2106/00004623-199904000-00009	1999	175	1,090
Fountas Kn, 2007, Spine	10.1097/BRS.0b013e318154c57e	2007	63	612
Mummaneni Pv, 2007, J Neurosurg-Spine	10.3171/spi.2007.6.3.198	2007	73	424
Kaiser Mg, 2002, Neurosurgery	10.1097/00006123-200202000-00001	2002	50	296
Katsuura A, 2001, Eur Spine J	10.1007/s005860000243	2001	33	294
Chiba K, 2006, Spine	10.1097/01.brs.0000250307.78987.6b	2006	11	278
Seichi A, 2001, Spine	10.1097/00007632-200103010-00010	2001	17	277
Pavlov H, 1987, Radiology	10.1148/radiology.164.3.3615879	1987	45	266
Ratliff Jk, 2003, J Neurosurg	10.3171/spi.2003.98.3.0230	2003	18	258
